# Management strategies in hospitals: scenario planning

**DOI:** 10.3205/iprs000065

**Published:** 2015-06-22

**Authors:** Mohamed Ghanem, Jörg Schnoor, Christoph-Eckhard Heyde, Sandra Kuwatsch, Marco Bohn, Christoph Josten

**Affiliations:** 1 Department of Orthopedic, Trauma and Plastic Surgery, University Hospital Leipzig, Leipzig, Germany; 2 Leipzig Graduate School of Management, Handelshochschule Leipzig HHL, Leipzig, Germany; 3 Department of Anesthesiology and Intensive Care Medicine, University Hospital Leipzig, Leipzig, Germany; 4 Human Resources and Legal Department, University Hospital Leipzig, Leipzig, Germany; 5 Department of Finance, Planning and Controlling, University Hospital Leipzig, Leipzig, Germany

## Abstract

**Background:** Instead of waiting for challenges to confront hospital management, doctors and managers should act in advance to optimize and sustain value-based health. This work highlights the importance of scenario planning in hospitals, proposes an elaborated definition of the stakeholders of a hospital and defines the influence factors to which hospitals are exposed to.

**Methodology:** Based on literature analysis as well as on personal interviews with stakeholders we propose an elaborated definition of stakeholders and designed a questionnaire that integrated the following influence factors, which have relevant impact on hospital management: political/legal, economic, social, technological and environmental forces.

These influence factors are examined to develop the so-called critical uncertainties. Thorough identification of uncertainties was based on a “Stakeholder Feedback”.

**Results:** Two key uncertainties were identified and considered in this study:

- the development of workload for the medical staff
- the profit oriented performance of the medical staff.

According to the developed scenarios, complementary education of the medical staff as well as of non-medical top executives and managers of hospitals was the recommended core strategy. Complementary scenario-specific strategic options should be considered whenever needed to optimize dealing with a specific future development of the health care environment.

**Conclusion:** Strategic planning in hospitals is essential to ensure sustainable success. It considers multiple situations and integrates internal and external insights and perspectives in addition to identifying weak signals and “blind spots”. This flows into a sound planning for multiple strategic options. It is a state of the art tool that allows dealing with the increasing challenges facing hospital management.

## Zusammenfassung

**Hintergrund:** Das Krankenhausmanagement ist stets Herausforderungen ausgesetzt. Anstatt auf die Herausforderungen zu warten, sollten Ärzte und Manager im Voraus handeln, um ein optimiertes und nachhaltig wertorientiertes Gesundheitswesen zu sichern. Diese Arbeit unterstreicht die Bedeutung der Szenarienplanung in Krankenhäusern, schlägt eine ausgearbeitete Definition der Stakeholder eines Krankenhauses vor und definiert die Einflussfaktoren, denen Krankenhäuser ausgesetzt sind.

**Methodik:** Basierend auf einer Literaturanalyse sowie auf persönlichen Interviews mit Stakeholdern eines Krankenhauses schlagen wir eine ausgearbeitete Definition von Stakeholdern vor und erarbeiteten einen Fragebogen. Dieser Fragebogen berücksichtigt folgende Einflussfaktoren, die entsprechende Auswirkungen auf das Krankenhaus-Management haben: politische/rechtliche, wirtschaftliche, soziale, technologische und Umweltkräfte.

Diese Einflussfaktoren werden untersucht, um die so genannten kritischen Unsicherheiten zu entwickeln. Die gründliche Identifizierung von Unsicherheiten basierte auf „Stakeholder Feedback“.

**Ergebnisse:** Zwei Haupt-Unsicherheiten wurden identifiziert und in dieser Studie berücksichtigt:

- die Entwicklung der Arbeitsbelastung für das medizinische Personal
- die gewinnorientierte Leistung des medizinischen Personals.

Entsprechend der entwickelten Szenarien konnten wir folgende Kernstrategie empfehlen: komplementäre Ausbildung sowohl des medizinischen Personals als auch der nicht-medizinischen Top-Führungskräfte und Manager von Krankenhäusern. Ergänzende szenariospezifische strategische Optionen sollen, falls erforderlich, in Betracht gezogen werden, um eine bestimmte zukünftige Entwicklung des medizinischen Umfeldes zu optimieren.

**Schlussfolgerung:** Strategische Planung in Krankenhäusern ist wichtig, um nachhaltigen Erfolg zu gewährleisten. Diese Planung soll mehrere Situationen berücksichtigen und integriert interne und externe Ein- und Ausblicke. Darüber hinaus soll sie schwache Signale und „blinde Flecken“ identifizieren. Dieses fließt in eine solide Planung für mehrere strategische Optionen ein. Es ist eine State-of-the-Art-Methode, die dem Krankenhausmanagement erlaubt, mit zunehmenden Herausforderungen umzugehen.

## Background

One of the most important factors that are increasingly considered by hospital business leaders and planners is uncertainty. The inability to adapt quickly enough to the rapidly changing business environment can be fatal for hospitals. These changes can be regarded as internal as well as external. This developing complex situation of the global health care business environment necessitates an even more thorough ability to examine the forces of change in order to find possible and plausible solutions to potential problems [4], [7], [10], [20], [25]. 

The goal of this work is to highlight the importance of scenario planning in hospitals, to propose an elaborated definition of the stakeholders of a hospital and to define and regroup the influence factors to which the stakeholders of the hospital and the hospital as an organization are exposed to. 

In many European countries, 21^st^ century high-tech medical service is delivered in hospitals and medical institutions, the majority of which is characterized by 19^th^ century organization structures, management methods and hierarchy [1], [3], [2], [6], [13], [17], [22]. Today, several strategies are adopted to improve health care service around the world [12]. In countries with a predominant statutory health care system such as Germany, attention is now paid to more patient autonomy, while in the USA the Patient Protection and Affordable Care Act known as “ObamaCare” attempts to provide more Americans with affordable quality health insurance [13], [15]. The health sector, no matter where, has become an enormously dynamic and complex market that also implies continued escalation in health spending and thus constitutes real challenges to hospital managers as well as to politicians [1], [6]. Therefore, we believe in the necessity of a change in mindset. Instead of waiting for challenges to confront us and then react, doctors and managers should act in advance to optimize and sustain value-based health [3], [5], [8], [16], [24]. 

## Methodology

Strategic planning must be applied to enable hospitals to quickly and flexibly adapt strategy to changes in the environment that become essential to their success [25]. To achieve this task, a modern strategic planning tool is needed with strategic planning processes offering the alignment and integration of external and internal perspectives enabling hospitals and managers to plan for multiple outcomes and options and therefore provides a sound basis for facing increasing challenges or future scenarios [25]. Several approaches to scenario planning have been developed since the method originated in the 1970 [11].

No detailed methodology to scenario planning is documented in literature. According to Chermack, this is the reason why only few understand the exact application methodologies [4]. Peter Schwartz called planning with scenarios “an art, not a science” [19]. Strategic planning is a well known method for coping with future changes in organizations. Strategic planning has yielded some insight about how organizations can predict and adapt to changes, yet it has not been really able to inform organization leaders about major political, environmental, economic and/or social changes [4]. Therefore, another school/approach to scenario planning has developed in which planners tell multiple stories that cover a variety of plausible future occurrences. This method opens eyes on a variety of possible futures [4], [7]. 

According to Schwartz, the scenario process entails thinking about the most remote and complex array of factors which affect any decision [19]. 

Schoemaker regards scenario planning as a tool that rather aims at challenging current paradigms of thinking and develop and anticipate a series of stories in which attention is directed to aspects that would have been otherwise overlooked [18].

Royal Dutch Shell defines scenario planning as “a method for acknowledging and working with what we don’t know and what we don’t know we don’t know” [20]. 

**Figure 1:**
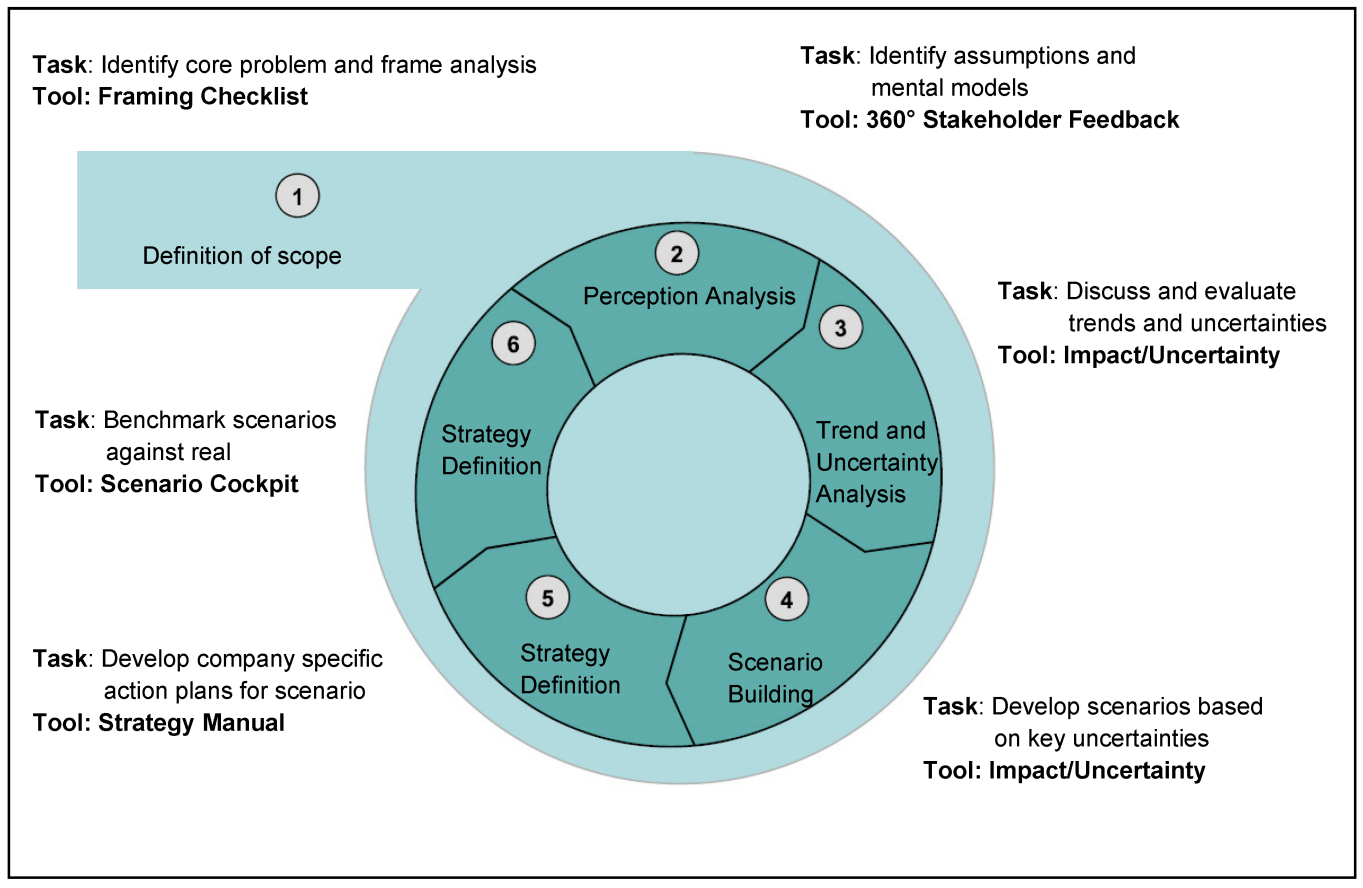
HHL-Roland Berger approach to scenario based strategic planning (Source: Wulf 2010 [25])

The most recent one was published in 2010 and is a standardized one with a structured procedure allowing a quicker and easier application in practice and reducing the complexity of the planning process [25]. Figure 1 (Fig. 1) illustrates the steps of this new approach.

All of the worldwide acknowledged methodologies integrate the following influence factors: political/legal, economic, social, technological and environmental forces [4], [18], [19], [21], [23], [25]. These influence factors are examined to develop the so-called critical uncertainties and predetermined elements [4], [18], [19], [21], [23], [25]. 

**Figure 2:**
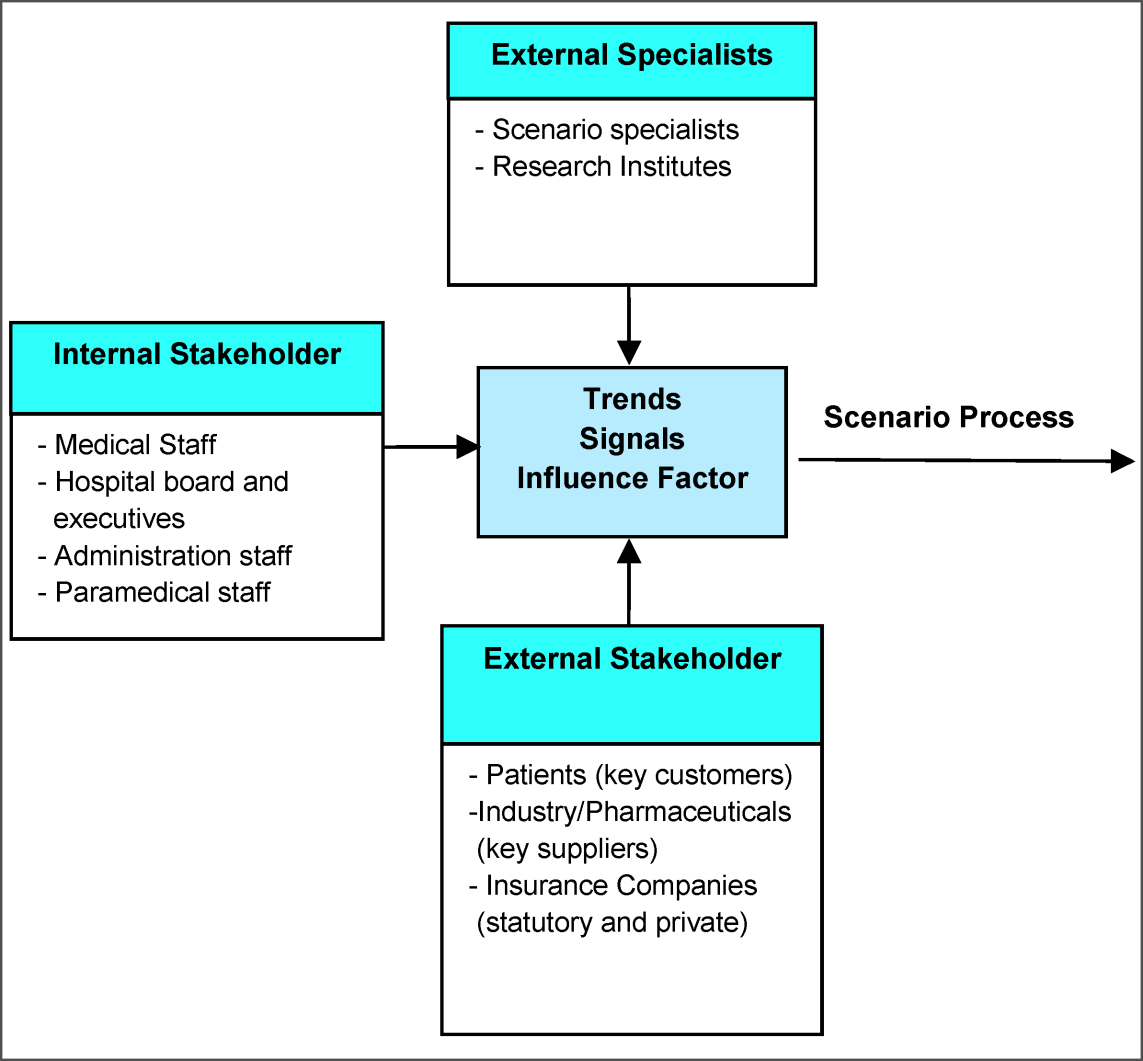
Feedback of the stakeholders of a hospital helps identifying trends, signals and influence factors (Adapted from Wulf 2010 [25])

Based on literature analysis [1], [3], [14], [22] as well as on personal interviews with stakeholders we propose an elaborated definition of stakeholders in Figure 2 (Fig. 2). 

Thorough identification of uncertainties must be based on a “Stakeholder Feedback” [25] (Table 1 (Tab. 1)), designed as a questionnaire that integrates the following influence factors: political/legal, economic, social, technological and environmental forces [4], [18], [19], [21], [23]. 

The condition sine qua none of strategic and scenario planning is the definition of scope and the identification of the influence factors to which hospitals are exposed. Again, based on literature analysis [1], [3], [2], [4], [14], [22], [25], and based on personal interviews with stakeholders, we integrated economic, social, political/legal, technological and environmental factors into the questionnaire. 

## Results

Over 100 questionnaires were sent to stakeholders. Over 60 were sent back to us in due time. After excluding incompletely filled questionnaires and those that were obviously filled out in an unserious manner, we analyzed 60 questionnaires representing samples of the stakeholders: medical staff (25%), executives and top management (5%), employees of the administration (10%), paramedical staff (15%), patients (25%), industry top management and management (5%), statutory and private insurance management (10%), external specialist (5%).

Scenarios were built upon the most uncertain developments and most important trends that have been identified, reflecting the most disruptive developments that can be currently imagined. Two key uncertainties were identified and considered in this study:

- the development of workload for the medical staff
- the profit oriented performance of the medical staff.

Both of these issues are social influence factors which have a huge economic impact on medical staff, on hospitals and on hospital executives and management as well as on the health sector as a whole. The multiple scenarios which were then developed are meant to inspire ideas and widen the vision of top management of hospitals to prepare for future challenges and to develop strategies and action plans that enable hospitals to remain competitive in a highly dynamic environment [25]. According to these multiple scenarios, proper and complementary education of the medical staff as well as of non-medical top executives and managers of hospitals is the recommended core strategy. Complementary scenario-specific strategic options should be considered whenever needed to optimize dealing with a specific future development of the health care environment [6]. 

By structuring the identified influence factors, separating environmental developments into predetermined and predictable trends as well as uncertainties and by determining uncertain driving forces, trend and uncertainty analysis builds the foundation for the consequent scenario development [25].

Whichever scenario planning method you employ, the scenarios developed are not intended to predict the future [4], [18], [19], [20], [21], [23], [25]. They are meant to inspire ideas and widen the vision of top management of hospitals to prepare for future challenges and to develop strategies and action plans that enable hospitals to remain competitive in a highly dynamic environment [25].

When analyzing and comparing the strategy implications for the scenarios developed, one can identify those recommendations that are common for all scenarios and those that are specific only for one or two of these scenarios. The common strategy recommendations build the basis of a core strategy and pave the way for being prepared to any of the predicted scenarios. This core strategy is applicable in all scenarios and should be complemented by scenario-specific strategic options whenever these are needed to optimize dealing with a specific future development of the health care environment [6], [25].

## Conclusion

Strategic planning in hospitals is essential to ensure sustainable success. It considers multiple situations, favorable as well as unfavorable, and integrates internal and external insights and perspectives in addition to identifying weak signals and “blind spots” [25]. This flows into a sound planning for multiple strategic options with flexibility towards different time horizons. It is a state of the art tool that allows dealing with the increasing challenges of the business world. 

This is the proper shield that allows soundly facing the increasingly challenging environment of the health care sector in general and a hospital in particular. Detailed strategy recommendations, however, are best derived according to the specific situation of the hospital considered.

## Notes

### Competing interests

The authors declare that they have no competing interests.

### Author’s statement and acknowledgment

This work is adapted from the Master Thesis “Scenarios for the German health care system using the example of a University hospital” submitted for the partial fulfilment of the MBA-Degree in General Management at the HHL in 2010 and was supervised by Prof. Dr. Torsten Wulf and Philip Meißner, MBA, Leipzig Graduate School of Management, HHL.

## Figures and Tables

**Table 1:**
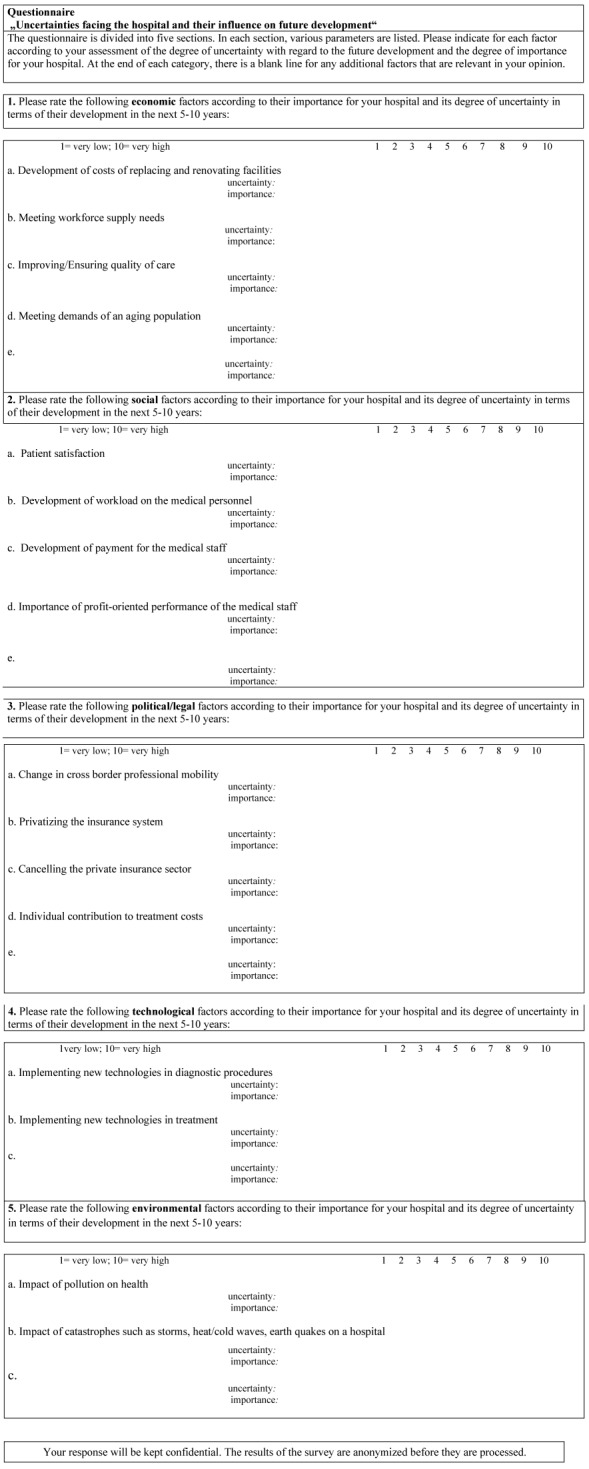
Questionnaire
